# Resting-State fMRI Functional Connectivity Is Associated with Sleepiness, Imagery, and Discontinuity of Mind

**DOI:** 10.1371/journal.pone.0142014

**Published:** 2015-11-05

**Authors:** Diederick Stoffers, B. Alexander Diaz, Gang Chen, Anouk den Braber, Dennis van ‘t Ent, Dorret I. Boomsma, Huibert D. Mansvelder, Eco de Geus, Eus J. W. Van Someren, Klaus Linkenkaer-Hansen

**Affiliations:** 1 Department of Integrative Neurophysiology, Center for Neurogenomics and Cognitive Research (CNCR), VU University Amsterdam, Amsterdam, The Netherlands; 2 Department of Sleep and Cognition, Netherlands Institute for Neuroscience, an institute of the Royal Netherlands Academy of Arts and Sciences, Amsterdam, The Netherlands; 3 Scientific and Statistical Computing Core, National Institute of Mental Health, an institute of the Department of Health and Human Services, Bethesda, Maryland, United States of America; 4 Department of Biological Psychology, Faculty of Psychology and Education, VU University Amsterdam, Amsterdam, The Netherlands; 5 Department of Medical Psychology, VU University Medical Center, Amsterdam, The Netherlands; Laureate Institute for Brain Research and The University of Oklahoma, UNITED STATES

## Abstract

Resting-state functional magnetic resonance imaging (rs-fMRI) is widely used to investigate the functional architecture of the healthy human brain and how it is affected by learning, lifelong development, brain disorders or pharmacological intervention. Non-sensory experiences are prevalent during rest and must arise from ongoing brain activity, yet little is known about this relationship. Here, we used two runs of rs-fMRI both immediately followed by the Amsterdam Resting-State Questionnaire (ARSQ) to investigate the relationship between functional connectivity within ten large-scale functional brain networks and ten dimensions of thoughts and feelings experienced during the scan in 106 healthy participants. We identified 11 positive associations between brain-network functional connectivity and ARSQ dimensions. ‘Sleepiness’ exhibited significant associations with functional connectivity within Visual, Sensorimotor and Default Mode networks. Similar associations were observed for ‘Visual Thought’ and ‘Discontinuity of Mind’, which may relate to variation in imagery and thought control mediated by arousal fluctuations. Our findings show that self-reports of thoughts and feelings experienced during a rs-fMRI scan help understand the functional significance of variations in functional connectivity, which should be of special relevance to clinical studies.

## Introduction

The investigation of the functional architecture of the human brain by means of resting-state functional magnetic resonance imaging (rs-fMRI) has rapidly become a major topic in neuroscience [1–7]. The analysis of temporal correlations between spatially distinct BOLD-signals in rs-fMRI data allows for quantification of functional connectivity [8] and the investigation of intrinsic connectivity networks (ICNs) [9]. Several ICNs have consistently been described [10, 11], along with changes in functional connectivity brought about by learning [12, 13], lifelong development [14, 15], brain disorders [16–20] or pharmacological intervention [21, 22]. However, some ICNs have been observed across widely different levels of consciousness [23–25], suggesting that coherent patterns of BOLD activity might not be closely coupled with conscious experiences during rest. Still, this leaves unanswered the fundamental question: how does functional connectivity within large-scale brain networks relate to conscious experiences in the resting state?

Exploring the relationship between functional connectivity and conscious experiences in the resting state is important, because in the absence of overt external task demands cognitive processes tend to become internally directed—comparable to what is commonly experienced during mind wandering [26]. The resting state may therefore be used as a model system for understanding mind wandering [27–29] whose neural correlates remain poorly understood [30, 31]. Furthermore, rs-fMRI is increasingly used to study systems-level mechanisms of brain disorders in which mind wandering could well be altered. For example, patients with affective disorders are known for their excessive worry and rumination [32, 33] and mind wandering increases when negative affect is induced [34]. Changes in thoughts and feelings are common also in neurological disorders such as Alzheimer’s disease and frontotemporal dementia [35, 36]. It remains unknown, however, whether putative changes in mind wandering associated with brain disorders will also be reflected in functional connectivity changes during rs-fMRI.

Different arguments support that BOLD-signal fluctuations during rs-fMRI could partially reflect neuronal activities governing conscious thoughts, for instance (1) BOLD-signal variation within ICNs is similar in magnitude to responses to stimuli in task-based fMRI [10], (2) their spatial patterns closely resemble networks engaged in stimulus-elicited cognition [37], and (3) given that there is no other source of conscious experience during rest—or mind wandering in general—than ongoing brain activity, it is plausible that measures derived from this activity, such as functional connectivity, would correlate with these experiences. Nonetheless, direct evidence for a relationship between ongoing brain activity and inner experiences appears limited [26, 38, 39], which could in part be due to a lack of validated instruments to quantify these experiences. To better capture multiple dimensions of thoughts and feelings that are characteristic of the mind-wandering-like experiences during the resting state, we have developed the Amsterdam Resting-State Questionnaire (ARSQ) [40, 41]. Equipped with the ARSQ, we here proceed to test the hypothesis whether self-report ratings of resting-state thoughts and feelings are related to measures of resting-state functional connectivity within ten large-scale functional brain networks in healthy participants.

## Materials and Methods

The study protocol was approved by the institutional review board of the VU University Medical Centre, Amsterdam, The Netherlands. All participants provided written informed consent prior to enrolment in the study.

### Participants

The current study was part of a larger study on sex differences in brain development that recruited individuals from the Netherlands Twin Register, a large database that contains longitudinal survey data from adult twins and their parents, siblings and spouses. In total, 106 self-declared healthy individuals participated in the present study; 1 monozygotic twin pair, 42 dizygotic opposite sex twin pairs, 4 dizygotic same sex twin pairs, 11 of their siblings and one unrelated individual from a pilot session; in the age range between 19 and 52 years (*M* ± *SD* = 30.2 ± 9.6 years; 53 females). Exclusion criteria were neurological disease and contraindications for MRI (e.g., pregnancy, ferromagnetic fragments, clips and devices in the body or claustrophobia).

### Data acquisition

Imaging was performed in a Philips Intera 3T MRI machine with an 8-channel head coil. For each participant, a high-resolution structural scan (3D T_1_-weighted gradient-echo sequence; 182 coronal slices, repetition time = 9.69 ms, echo time = 4.6 ms, flip angle = 8°, 182x256x256 matrix; voxel size = 1x1x1.2 mm) and two rs-fMRI scans (echo-planar imaging sequence; 140 volumes, repetition time = 2200 ms, echo time = 30 ms, flip angle = 80°, 80x80x38 matrix, voxel size = 2.75 mm isotropic with 10% inter-slice gap, ascending order, scan length = 5 minutes and 8 seconds) were acquired. Scans were separated by approximately 45 minutes of task-related fMRI. Participants received consistent instructions before (i.e., “The next scan takes about six minutes. It is important for this scan that you try to relax, lie still with your eyes closed, and try not to fall asleep.”) and after (i.e., “You will now see a number of statements about possible feelings, sensations and thoughts that you might have experienced during the scan. Please indicate to what degree you concur with each statement.”) each rs-fMRI scan. During the 45 minutes in between two runs of resting-state fMRI participants performed the Tower of London task (probing planning and working memory) and the Stroop task (probing selective attention and processing speed).

### Self-reports of resting-state cognition

**Fig 1:**
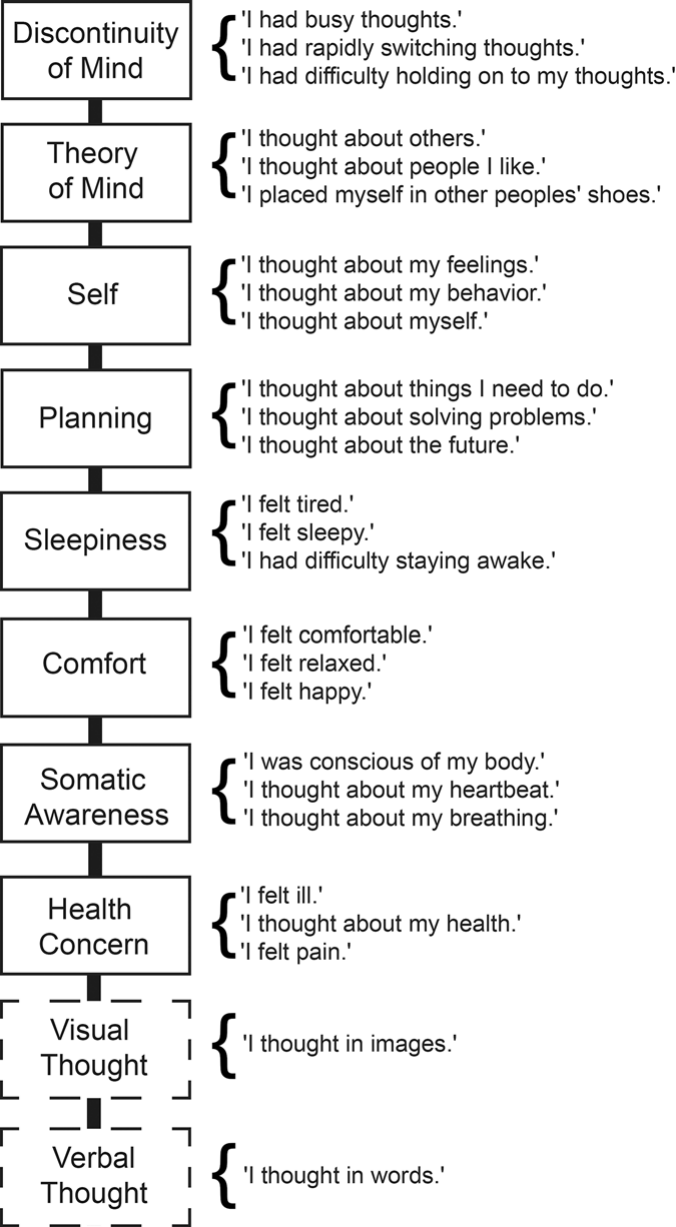
A resting-state experience can be partitioned into multiple dimensions. The different dimensions of the resting-state mind-wandering experience as probed by the Amsterdam Resting-State Questionnaire are shown in black boxes. Dimensions sampled by single items are shown in dashed boxes, items used to model each of these dimensions are shown to the right of each box.

To quantify thoughts and feelings during the resting-state session, participants completed a computerized version of the Amsterdam Resting-State Questionnaire (ARSQ: [40]) immediately following each block while still in the MRI bore. The ARSQ is a list of Likert-type statements rated on a scale from 1 (“Completely disagree”) to 5 (“Completely agree”) probing potential subjective experiences arising during the resting state in an efficient manner, minimizing potential bias related to recollective ability [3]. Based on a large collection of ARSQ data, a revised and extended 10-factor model of mind-wandering experiences during the resting-state has been derived (Fig 1: [41]), allowing for the quantitative assessment of ten dimensions of mind wandering, by averaging the ratings of the ARSQ statements within each factor. Because part of the present data were acquired prior to the revision of the ARSQ [41], the dimensions “Verbal Thought” and “Visual Thought are only represented by the single statements “I thought in words” and “I thought in images”, respectively. The average time to complete the ARSQ in this setting was stable across the two trials (trial 1: 216 ± 40 seconds, range 129–346; trial 2: 221 ± 45 seconds, range 135–362; *t*(105) = -1.067, *P* = .29). To avoid potential (recollection) bias, the ARSQ item order was randomized across trials and participants.

### MRI pre-processing

Imaging data were pre-processed using FSL (FMRIB's Software Library [42, 43], version 4.1.8, Analysis Group, FMRIB, Oxford, UK). Acquisition did not include field maps. Pre-processing included motion correction, removal of non-brain tissue, spatial smoothing using a 5 mm full-width-at-half-maximum Gaussian kernel, high-pass temporal filtering equivalent to 100 seconds and fully affine linear registration to standard (Montreal Neurological Institute) space at 2 mm isotropic resolution. Slice timing correction was not applied, as effects on ICNs are expected to be negligible (most of the power in ICNs lies at lower frequencies, also see [44], and the procedure can have adverse effects, e.g., spreading the effect of potential artefacts or outlier scans). In addition, single-session ICA was performed to prepare data for FMRIB's ICA-based Xnoiseifier (FIX) [45, 46] to denoise the rs-fMRI data (e.g. regress out components associated with movement, cardiac pulsation and arterial contribution, large veins, MRI acquisition/reconstruction or susceptibility motion). Next, FIX was trained using datasets of which ICA components had manually been classified into “good” and “bad” components by one of the experimenters (DS; see S1 Fig for examples of components classified as artefactual or signal). The training sample consisted of the first rs-fMRI session of 15 participants that were selected out of the total of 106 participants on the basis of their Netherlands Twin Registry database code (in ascending order starting at the lowest number) and had been scanned at different times throughout the duration of the study. Next, FIX denoising was run on all datasets, resulting in preprocessed rs-FMRI data from which the majority of movement, cardiac, respiratory and scanner noise has been removed.

### ICN selection

After single-session ICA and FIX denoising, ICA with principal component analysis-driven automatic dimensionality reduction [11] was performed on all concatenated datasets using FSL’s MELODIC, yielding 20 different components. Next, we used spatial correlation to identify those components that best matched the spatial maps derived from an ICA on BrainMap task-based fMRI activation maps [37]. These maps have the advantage of being very robust (derived from fMRI experiments in nearly 30,000 human subjects), they are publicly available (http://fsl.fmrib.ox.ac.uk/analysis/brainmap+rsns/) and have been mapped onto “behavioral domains” (experimental paradigm classifications) in the BrainMap database [47]. As we had only limited coverage of the cerebellum, the BrainMap cerebellar activation map was excluded. Eight ICA spatial maps could be readily classified into Medial Visual, Occipital Visual, Lateral Visual, Sensorimotor, Auditory, Executive, Left Fronto-Parietal, and Right Fronto-Parietal ICNs. Two spatial maps showed high spatial correlations (Pearson *r* = .45 & .51) with the default mode ICN, giving a total of 10 ICNs to be associated with ARSQ scores. The weakest spatial correlation in the 10 paired maps is *r* = .25. A correction for the spatial degrees of freedom is given via Gaussian random field theory and empirical smoothness estimation [48, 49]; for this data, the number of independent ‘‘resels” (resolution elements) was found to be 703; we therefore set this conservatively to be 500. A two-sided correction for the number of possible paired comparisons would be a factor of 10 * 10 * 2 = 200. Applying Fisher’s *r*-to-*Z* transform by using degrees-of-freedom 500, converting to a *P*-value and multiplying by 200 to correct for multiple comparisons, we obtain P = 2.5 * 10^−6^, hence, the weakest pairing corresponds to (at least as small as) P = 10^−5^ (corrected).

### Estimation of voxel-wise functional connectivity

For each session, voxel-wise estimates of functional connectivity were obtained using a regression technique referred to as the “dual-regression” approach [50, 51]. The selected ten spatial maps from our ICA analysis were used as a set of spatial regressors in a general linear model to find the average time course of the BOLD signal within each of the ten maps. Next, the resulting time courses were variance-normalized and used as a set of temporal regressors in a general linear model to find session-specific spatial correlation maps (still associated with the group-level maps). After this dual regression, the resulting spatial maps of voxel-wise functional connectivity were collected into single 4-dimensional files for each of the ten selected ICA components (i.e., the fourth dimension contains the 2 x 106 session-specific maps of functional connectivity). Voxel values in the spatial correlation maps reflect both ICN amplitude and correlation strength with the average (normalized) ICN time course in (globally normalized) “BOLD” units.

To investigate whether our results can be generalized to ICNs defined in an independent dataset, we repeated dual regression using the nine spatial maps derived from an ICA on BrainMap task-based fMRI data in a separate analysis.

### Linear mixed-effects modeling

To investigate the association between an ARSQ measure and functional connectivity while properly taking into account both correlations among sessions from the same participant and the family relationship of our subjects (twins and siblings), we implemented a linear mixed-effects (LME) model. The ten 4-dimensional files containing voxel-wise functional connectivity estimates were analyzed in an LME model for each of the 10 ICA-derived ICN components and each ARSQ measure separately. The fixed effects were the intercept and the association between the ARSQ measure and functional connectivity, while the random effects included how families deviated from overall intercepts and slopes. The LME framework allowed us to specify a generic correlation structure among the random effects and account for the correlations among sessions from the same participant as well as among twins and siblings from the same family. Analyses were performed using the 3dLME software tool [52] in AFNI (Analysis of Functional NeuroImages, Scientific and Statistical Computing Core, NIMH, Bethesda, MD) [53]. The association between an ARSQ measure and functional connectivity was the effect of interest. Voxel-wise significance was set at a threshold of *P <* .01 (one-tailed) and corrected for family-wise error (FWE) within the spatial mask using Gaussian random field theory at a cluster-wise Bonferroni-corrected (2 directions * 10 ICNs * 10 ARSQ factors) significance threshold of *P*_*FWE*_ *<* .00025 through Monte Carlo simulations using the 3dClustSim tool in AFNI. To derive a functionally relevant measure of functional connectivity from each ICN and each session, the clusters that showed a relation of functional connectivity with ARSQ measures were used to extract mean functional connectivity values from each individual spatial map using FSL tools. This mean functional connectivity value is used for scatter plots to illustrate the associations with the different ARSQ factors. In addition to the ICA-derived masks, linear mixed-effects modeling was performed using BrainMap masks.

## Results

**Fig 2:**
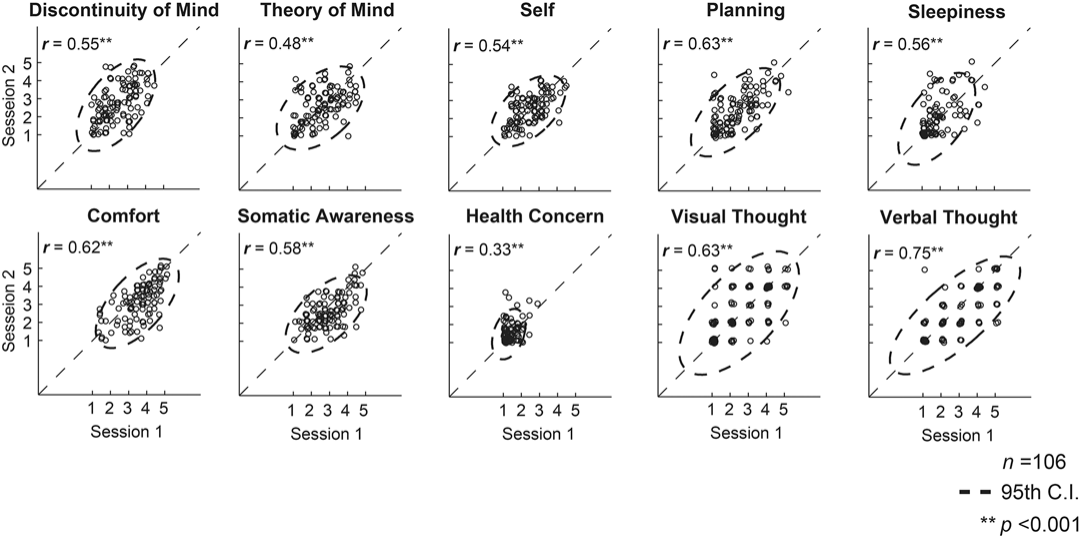
Mind wandering during a resting state primarily varies across subjects. The mind-wandering experiences over two resting-state sessions were strongly and positively correlated (Pearson), yet displayed substantial between-subject variation. To aid visualization, jitter (*M* ± *SD* = 0.1 ± 0.05) was added to the data points; dashed ellipses indicate the 95% confidence limit, statistics describe raw data before adding jitter.

**Fig 3:**
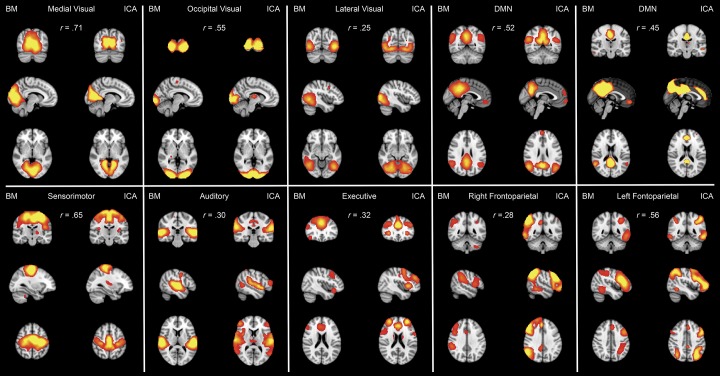
BrainMap components are well matched to ICA components. Ten ICNs from the analysis of the 29,671-subject BrainMap activation database (Right column of each pair: [37]) paired to 10 ICNs that were selected out of 20 components from the ICA analysis of the current rs-fMRI dataset (right column). The networks were paired using spatial cross-correlation with mean *r* = .46 (range = .25–.71); the weakest of these correlations thus has a significance of *P* = 10^−5^ (corrected). All ICA spatial maps were converted to *Z* statistic images via a normalized mixture–model fit, and then thresholded at *Z* = 3. The figure shows the 3 most informative orthogonal slices for each pair. ICNs are displayed in a gradient from red to yellow (3 < *Z* < 5), superimposed on the MNI152 standard space template image, in radiological convention (left = right). The *r* statistic is displayed at the top of each panel.

The ARSQ [40] was completed immediately after each session of eyes-closed rs-fMRI while still lying in the bore and, subsequently, scores on ten dimensions of resting-state cognition were computed (see Methods and Fig 1). ARSQ scores between the two sessions of rs-fMRI were all positively and significantly correlated (.33 ≤ *r* ≤ .75, *P* < .001) and also exhibited substantial between-subject variation along the rating scale as can be seen from Fig 2. Using dual-regression [50, 51], we obtained functional connectivity values for each individual session of a subject for both the ten well-matched pairs of components from our ICA analysis as well as nine (i.e., excluding the cerebellar ICN) from the analysis of functional fMRI data by Smith and co-workers [37]. The ICN maps as well as their spatial correlations are shown in Fig 3. In the following, we evaluate voxel-wise associations of resting-state cognition with functional connectivity values (see Methods).

### Association between ICA-based ICNs and resting-state cognition

**Fig 4:**
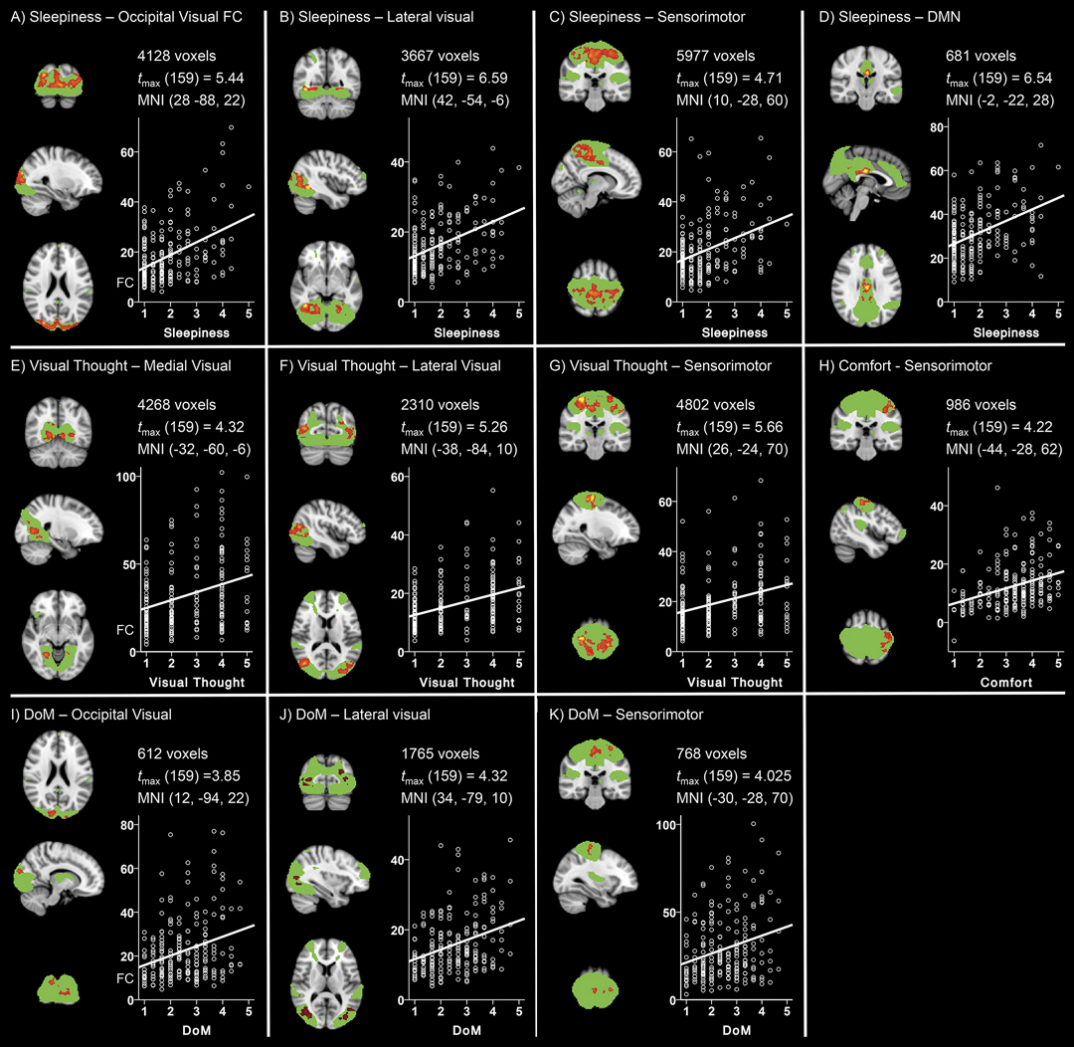
Sleepiness promotes widespread increases in functional connectivity. A total of eleven combinations of ICNs and ARSQ dimensions showed significant associations between functional connectivity and ARSQ score at a cluster-wise Bonferroni-corrected significance threshold of *P*_*FWE*_ = .00025. Orthogonal slices through the peak voxel are shown on the left of each panel; ICNs are defined by the ICA spatial maps thresholded at *Z* = 2.3 and displayed in green, clusters of voxels showing significant associations (2.62 < *t* < 6) in a gradient from red to yellow, overlaid on a standard brain template in radiological convention (left = right). Number of significant voxels, maximum *t*-statistic and peak voxel coordinates are displayed at the top right, scatter plots showing the mean functional connectivity value in significant clusters (y-axis) plotted against the relevant ARSQ measure are shown in the bottom right of each panel.

A total of four dimensions of resting-state cognition and five different ICNs showed positive associations at a Bonferroni corrected cluster-wise significance threshold of *P*_*FWE*_ = .00025 (Fig 4). Subjective ratings of sleepiness exhibited the most pronounced associations with higher scores characterized by increased functional connectivity in widespread regions within the Occipital Visual, Lateral Visual, Sensorimotor and Default Mode networks (Fig 4A–4D). These findings are in line with recent EEG-fMRI work showing increased BOLD functional connectivity in occipital brain regions with EEG-defined decrements in arousal [4].

Interestingly, we observed positive associations between Visual Thought and functional connectivity within the Medial Visual, Lateral Visual and Sensorimotor networks (Fig 4E–4G), i.e., a set of patterns very similar to those affected by sleepiness (Fig 4A–4D). Moreover, Visual and Sensorimotor networks also exhibited positive associations with Discontinuity of Mind (Fig 4I–4K). The items used for probing Sleepiness, Visual Thought and Discontinuity of Mind are very different (cf. Fig 1); however, inter-factor correlation analysis indicated that these dimensions are related (Table 1). For example, Sleepiness correlates positively with Discontinuity of Mind—as one would expect [54]—and Discontinuity of Mind in turn correlates positively with Visual Thought. Finally, Comfort showed significant positive associations with functional connectivity within the Sensorimotor network (Fig 4H).

**Table 1:** Interfactor correlation matrix of ARSQ dimensions (Pearson correlation).

*N* = 106	DoM	ToM	Self	Planning	Sleepiness	Comfort	Somatic Awareness	Health Concern	Visual^1)^ Thought	Verbal^1)^ Thought
Discontinuity of Mind (DoM)	-									
Theory of Mind (ToM)	.32*	-								
Self	.43*	.32*	-							
Planning	.33*	.45*	.38*	-						
Sleepiness	.24*	.10	-.02	.09	-					
Comfort	-.13	.13	-.12	.14	.04	-				
Somatic Awareness	.11	.01	.32*	.13	-.11	-.06	-			
Health Concern	.08	.04	.26*	.15	.02	-.26*	.20	-		
Visual Thought	.26*	.37*	.18*	.23	.12	.24*	.02	-.09	-	
Verbal Thought	-.01	.25*	.26*	.16*	-.04	.07	.13	.05	-0.18*	-

1) These dimensions were based on single items only.

*) *P*_*FDR*_ < .05

### Association between BrainMap ICNs and resting-state cognition

**Fig 5:**
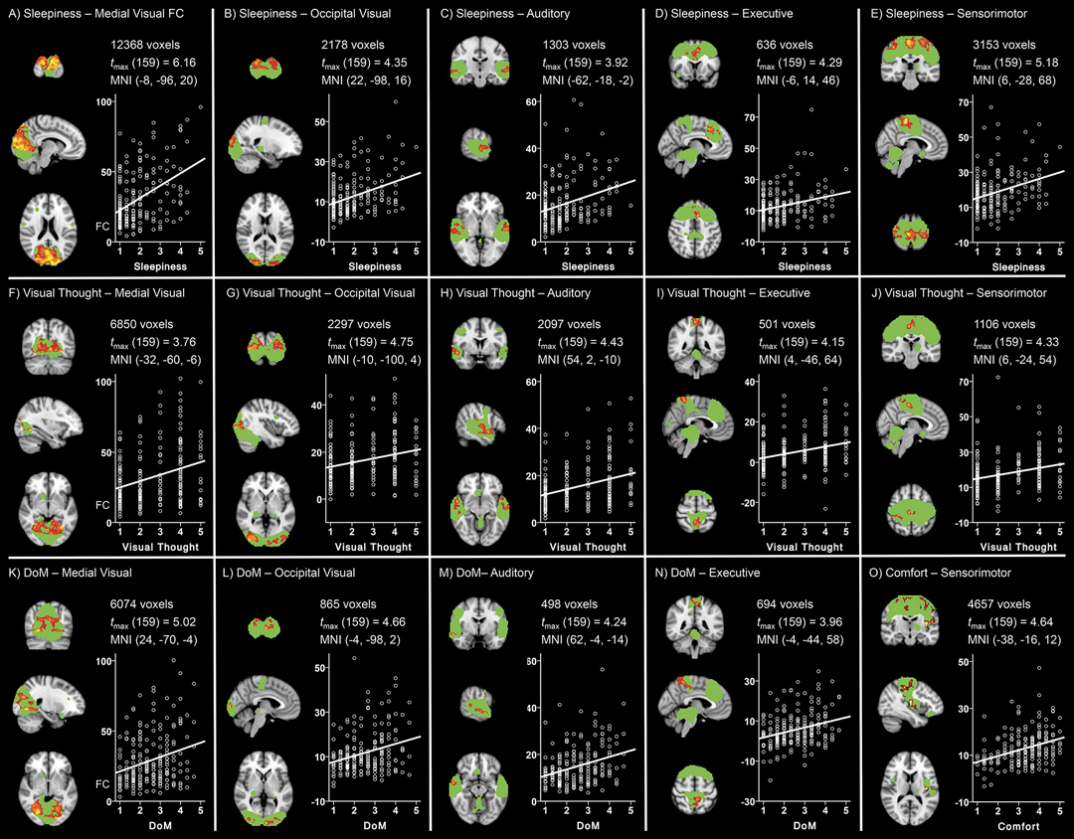
BrainMap-derived ICNs show similar associations with ARSQ scores to those of the ICA maps. A total of fifteen combinations of ICNs and ARSQ dimensions showed significant associations between functional connectivity and ARSQ factor score at a cluster-wise Bonferroni-corrected significance threshold of *P*_*FWE*_ = .00025. Orthogonal slices through the peak voxel are shown on the left of each panel; ICNs are defined by the BrainMap spatial maps thresholded at *Z* = 2.3 and displayed in green, clusters of voxels showing significant associations (2.62 < *t* < 5) in a gradient from red to yellow, overlaid on a standard brain template in radiological convention (left = right). Number of significant voxels, maximum *t*-statistic and peak voxel coordinates are displayed at the top right, scatter plots showing the mean functional connectivity value in significant clusters (y-axis) plotted against the relevant ARSQ measure are shown in the bottom right of each panel.

All of the selected ICA-derived spatial maps in the previous paragraph had a spatial correlation above *r* = .25 with ICNs derived from the BrainMap database (Fig 3). To test the robustness of our results against the definition of the ICNs, the linear mixed-effects modeling was also performed using the BrainMap masks. Positive associations at a Bonferroni corrected cluster-wise significance threshold of *P*_*FWE*_ = .00025 were observed between four dimensions of thoughts and feelings probed by the ARSQ and five different ICNs (Fig 5). In agreement with the ICA-based analysis, subjective ratings of sleepiness exhibited the most pronounced associations with higher scores characterized by increased functional connectivity in widespread regions within the Medial Visual, Occipital Visual, Auditory, Executive Control and Sensorimotor networks (Fig 5A–5E). These findings are remarkably similar to the increased BOLD functional connectivity in occipital brain regions, auditory cortices, and the executive control network observed with EEG-defined decrements in arousal [4].

Visual Thought showed positive associations with functional connectivity within the Medial Visual, Occipital Visual, Auditory, Executive Control and Sensorimotor, networks (Fig 5F–5J), i.e., a set of patterns very similar to those affected by sleepiness (Fig 5A–5E) and those of ICA-derived masks (Fig 3E–3G). Interestingly, these regions—except for the sensorimotor network—also exhibited positive associations with Discontinuity of Mind (Fig 5K–5N). As noted above, however, this may in part be explained by the significant inter-factor correlations of these dimensions (Table 1). Finally, Comfort showed significant positive associations with functional connectivity within the Sensorimotor network as defined also by the BrainMap mask (Fig 5O).

Together, the present findings suggest that arousal fluctuations have pronounced effects on fMRI functional connectivity and that subjective self-reports can reveal these effects as well as their implications for imagery and thought control, both using ICNs derived from an ICA on the present data and ICNs derived from an ICA analysis on fMRI activation maps.

## Discussion

Interest in resting-state brain activity has grown explosively over the past 15 years and challenged our view on the functioning of the human brain [55, 56]. The overwhelming majority of the brain’s activity is not driven by external stimuli and is organized into complex spatio-temporal patterns whose quantitative character and functional role we are only beginning to comprehend [5–7]. To our knowledge, this is the first investigation of the relationship between functional connectivity within multiple ICNs and subjective experiences during an rs-fMRI scanning session. Our data indicate that the strength of functional connectivity during wakeful rest is functionally important. In particular, individual variation in perceived Sleepiness exhibited pronounced and positive associations with functional connectivity in 5 of the 10 ICNs investigated (Fig 3). Importantly, increasing imagery or discontinuity of mind—possibly associated with sleepiness—pointed to enhanced functional connectivity in very similar brain regions. Our results are in line with recent studies investigating the relationship between EEG-defined arousal and BOLD functional connectivity [4] and highlight the potential benefits of assessing thoughts and feelings experienced during rs-fMRI for a better interpretation of differences in functional connectivity, e.g., when used as a biomarker in clinical research [57] or to assess the effects of intervention [58].

### Dynamics of arousal and functional connectivity in resting states

The resting-state condition, when being used for neurophysiological assessments, has recently received renewed criticism for assuming 1) that subjects think about the same things and 2) that their overall brain state is constant throughout the measurement [4]. EEG in combination with fMRI offers a powerful means to test the second assumption. EEG-fMRI has shown that up to one third of normal subjects tend to transition from wake to stage-1 sleep within the first 3 minutes of an eyes-closed rs-fMRI scanning session and that this is associated with increased BOLD functional connectivity especially in occipital brain regions, auditory cortices, and the executive control network [4]. These findings are well-supported, as drowsiness is associated with increased theta oscillation amplitude while alpha oscillation drop out, and both of these electrophysiological markers have been associated with increased functional connectivity in several ICNs including visual and sensorimotor networks [59–61]. In addition, the dorsal attention network is known to increase its functional connectivity when subjects enter light sleep compared to wakefulness [24]. Our data confirm these findings, with 15% of participants scoring high on sleepiness (> 3 on the 5-point scale), while also showing that even a quantitative self-report of sleepiness based on three simple statements is sufficient to observe these associations. Alternatively, opening or closing the eyes could influence activity and connectivity in visual and sensorimotor networks; however, more than 94% of subjects reported having their eyes closed in both sessions (rating agree to completely agree), which in our opinion makes it unlikely that visual input or eye open/closure biased the associations between the ARSQ and functional connectivity.

We have previously observed that ARSQ scores on Sleepiness correlate positively with clinical measures of insomnia, anxiety, and depression [40]. As network analyses of the associations between psychopathological symptoms have identified insomnia symptoms as a key connecting symptom [62], psychiatric patients are likely to show elevated Sleepiness during resting-state neuroimaging. Thus, considering the many networks exhibiting increased functional connectivity with sleepiness in the present study, one should be careful in interpreting such differences in terms of a primary pathophysiology without complementary data on Sleepiness. Ideally, control and patient groups would be matched for arousal and sleepiness and the ARSQ score could prove valuable in this regard.

### Mind wandering and resting-state fMRI

Considering the importance of the resting state in neuroimaging and the critical role of ongoing brain activity for the emergence of inner experiences, strikingly little has been done to investigate this relationship. As of yet, we are aware of only three studies [26, 38, 39]. This apparent scarcity may partly be due to the lack of validated instruments to quantify thoughts and feelings during the resting-state scan [27], and partly due to the widespread view that the resting state is an instruction “to do nothing” [1] or a “no task” condition [63], something which has been suggested to impose insufficient constraints on cognition to render rs-fMRI patterns interpretable [64]. In line with a previous report [65], our data suggest that people are indeed differentially engaged in cognitive processes during the resting state [40], but also that the large variation in the content and quality of mind wandering between participants can be captured by a validated tool such as the ARSQ (Fig 2, [41]). Given this degree of variation, the modest, yet significant and positive ARSQ-score correlations between the two resting-state sessions should not be viewed as indicating a lack of validity. Instead, they suggest that aspects of resting-state cognition, despite their inherent volatility, exhibited a substantial amount of intra-individual stability over the course of our experiments, which is in agreement with test-retest correlations observed on time scales of months [41].

A pioneering study on fMRI activation patterns and inner experiences during the resting state, focusing specifically on DMN connectivity and self-reports, identified a positive correlation with past and future thoughts [26]. However, the differences in fMRI analysis methodology as well as the approach with which experiences were quantified differed substantially from the present study, making it difficult to draw comparisons. For the DMN, we only observed a significant association with Sleepiness and the ARSQ does not specifically quantify the fraction of time spent thinking about the past or future. Similar comments apply to a more recent study in which correlations between—as opposed to within—ICNs were linked to scores on imagery and inner speech obtained with a semi-structured interview rather than a self-report scale like the ARSQ [38]. Although correlations between ICNs could also be quantified in the present study, the number of possible combinations is large (*n* = 45) and testing all these combinations for their associations with the 10 ARSQ dimensions would require Bonferroni correction for the additional 450 tests. We would not expect our study to have the power to support such stringent statistical correction and will leave this option for future investigation. A recently developed self-report questionnaire is more similar to the ARSQ as statements related to thought content and form were rated on a standardized (9-point) scale [39]. Still, it is difficult to compare the present results to imaging results obtained using their questionnaire, because the fMRI was analyzed using measures of Fractional Amplitude of Low Frequency Fluctuations, Regional Homogeneity, and Degree Centrality as opposed to our dual-regression estimate of functional connectivity in large-scale brain networks. In addition, their questionnaire did not probe feelings such as Sleepiness and Comfort, which demonstrated the strongest associations with functional connectivity in our study. We also note that their data on thoughts referred to an hour-long scanning session and the questionnaire was completed more than 20 minutes after the rs-fMRI scanning session. It is our experience that the memory of feelings—and especially thoughts—quickly fade away and, therefore, we would recommend future studies to use a standardized questionnaire that can be completed inside the scanner directly after concluding the rs-fMRI scanning session [41].

All of the studies that have used retrospective scoring of mind wandering during rs-fMRI—including the present one—have included more than 100 participants in the analysis. From this perspective, one may consider the number of associations between thoughts, feelings and BOLD signals surprisingly low. In our case, 10 ICNs and 10 dimensions of resting-state cognition could have revealed 200 significant effects when including also the possibility of negative associations. After Bonferroni correction, however, only 11 and 15 associations remained significant for the ICA and BrainMap masks, respectively. Apart from the conservative statistical approach, important factors contributing to a limited number of significant associations presumably include the very large brain regions used to assess the functional connectivity compared to the anticipated greater functional-anatomical specialization involved, e.g., in specific acts of Theory of Mind, Self- or Planning-related processing, thereby favoring dimensions in the ARSQ that index general changes in brain state. Furthermore, over the course of a 5-minute scanning session, both the content and quality of mind wandering—as well as the functional connectivity may vary greatly [66], and the present analysis is based on an average estimate of both. Another explanation that should be mentioned is that functional connectivity as measured using the low temporal resolution offered by current rs-fMRI protocols may simply not be strongly related to the contents and quality of thought [2]. In spite of aforementioned shortcomings, and given the limited time needed to gather ARSQ data in the scanner and the growing use of rs-fMRI, we still recommend collecting subjective self-reports. Large-scale databases may well be the key to the detection of less pronounced associations that nonetheless could represent important functional-anatomical templates for interpreting variation in rs-fMRI signals in a wide variety of settings. For example, it is remarkable that we did not observe any significant negative associations. Although negative associations were found at the voxel level, most notably for frontoparietal ICNs and ToM, Self, and Verbal thought, these did not survive the present conservative Bonferroni-corrected cluster-size thresholds. Large-scale rs-fMRI and ARSQ databases could also foster the development of new analysis tools and experimental paradigms, such as the real-time decoding of rs-fMRI [4, 67, 68].

### Information processing and functional connectivity

Remarkably few fMRI studies have explicitly addressed the relationship between brain activation (i.e., BOLD responses) and functional connectivity (temporal correlations between BOLD signals). A positive association may seem natural to assume, because coherent fluctuations are best detected with high signal-to-noise ratio BOLD fluctuations. However, different lines of evidence suggest an alternative model. For example, during natural viewing conditions, functional connectivity decreased markedly between non-homologous visual areas during visual stimulation relative to rest, which was interpreted as decoupling between visual areas that engage in specialized processing [69]. Similarly, Nir et al. reported a marked spatial de-correlation in visual cortices while BOLD variance increased when subjects viewed a film as opposed to fixation [70]. Likewise, when comparing the variance of spatial modes of activity fluctuations in visual areas V1-V4 during motion-induced blindness, local, retinotopically specific fluctuations were linked to the perceptual dynamics, whereas the spatially non-specific fluctuations were not, even though the latter accounted for a greater proportion of the BOLD-signal variance [71]. These findings indicate that spatio-temporal differentiation of BOLD activity during a constant-stimulus condition is important for specific, intrinsically generated perceptual events. Concertedly, these studies suggest that BOLD responses and temporal correlations between BOLD signals may be inversely related—or unrelated. In line with this, we speculate that disengaging from focused internal mentation due to drowsiness is accompanied by widespread coherent and non-specific hemodynamic oscillations within ICNs and a corresponding increase in functional connectivity. One may regard the positive correlations between Visual Thought and functional connectivity in Medial Visual and Occipital Visual networks as exceptions to this framework; however, we suggest that imagery has a less strong de-correlating effect compared to processing visual stimuli and that imagery effects are overshadowed by the pronounced increases in functional connectivity related to Sleepiness and the positive correlation between Visual Thought and Sleepiness (Table 1). As more data become available, future studies could consider separately analyzing associations between rs-fMRI and ARSQ scores from subgroups of subjects defined by, e.g., Sleepiness scores in the range of 1–2 (strongly disagree and disagree) to limit the confounding effect on other dimensions of mind wandering. We also urge that the relationship between BOLD activation and functional connectivity is better investigated, e.g., by applying functional connectivity analysis to task-based fMRI data.

### Implications for biomarker development

Resting-state neuroimaging features prominently in the clinical literature (for reviews, see [17, 18, 19]). Little has been done, however, to characterize the thoughts and feelings experienced by patients during resting-state scanning. We identified numerous associations between different dimensions of resting-state cognition during rest and functional connectivity in ICNs, suggesting that the use of questionnaires such as the ARSQ is instrumental if one aims to explore the extent to which variation in functional connectivity is related to variation in non-sensory experiences [3], which may vary systematically in development and aging [14, 15], brain disorders [16–19], pharmacological intervention [21, 22], or learning [12, 13]. We propose that our understanding of neural correlates of thoughts and feelings in the resting state can be brought much further with larger sample sizes—which has fueled much of the progress in the field of neuroimaging [37, 72–74]—and by exploring the rich variety of biomarker algorithms applicable to resting-state neuroimaging.

## Supporting Information

S1 Fig Artefactual and signal components. Examples of “good” (panels A, B and C) and “artefactual” (panels D, E and F) components for the first participant. For good components, spectral power lies primarily between 0 and 0.05 Hz and the signal above threshold follows cortical gyrification. For movement-related artefacts, the signal above threshold is essentially at the edges of the brain and the frequencies of the power spectra are disparately distributed. In components due to cardiac pulsation and arterial contribution, the signal above threshold in the spatial maps is essentially located in the ventricles, or following the main arteries (posterior cerebral artery, middle cerebral branches). For components relating to large veins, the signal above threshold in the spatial maps is essentially following the sagittal sinus. Additionally, one may find artefactual components due to MRI acquisition/reconstruction, limited to the white matter or due to susceptibility-motion. For a detailed description of ICA-based artifact removal, see [45, 46].
(PDF)
