# Supplementary material for: Resting-State fMRI Functional Connectivity Is Associated with Sleepiness, Imagery, and Discontinuity of Mind
Source: PLoS One. 2015 Nov 5;10(11):e0142014. doi: 10.1371/journal.pone.0142014 (PMC4634926; doi:10.1371/journal.pone.0142014)

Fig. S1a. Signal ICA component (Occipital Visual)

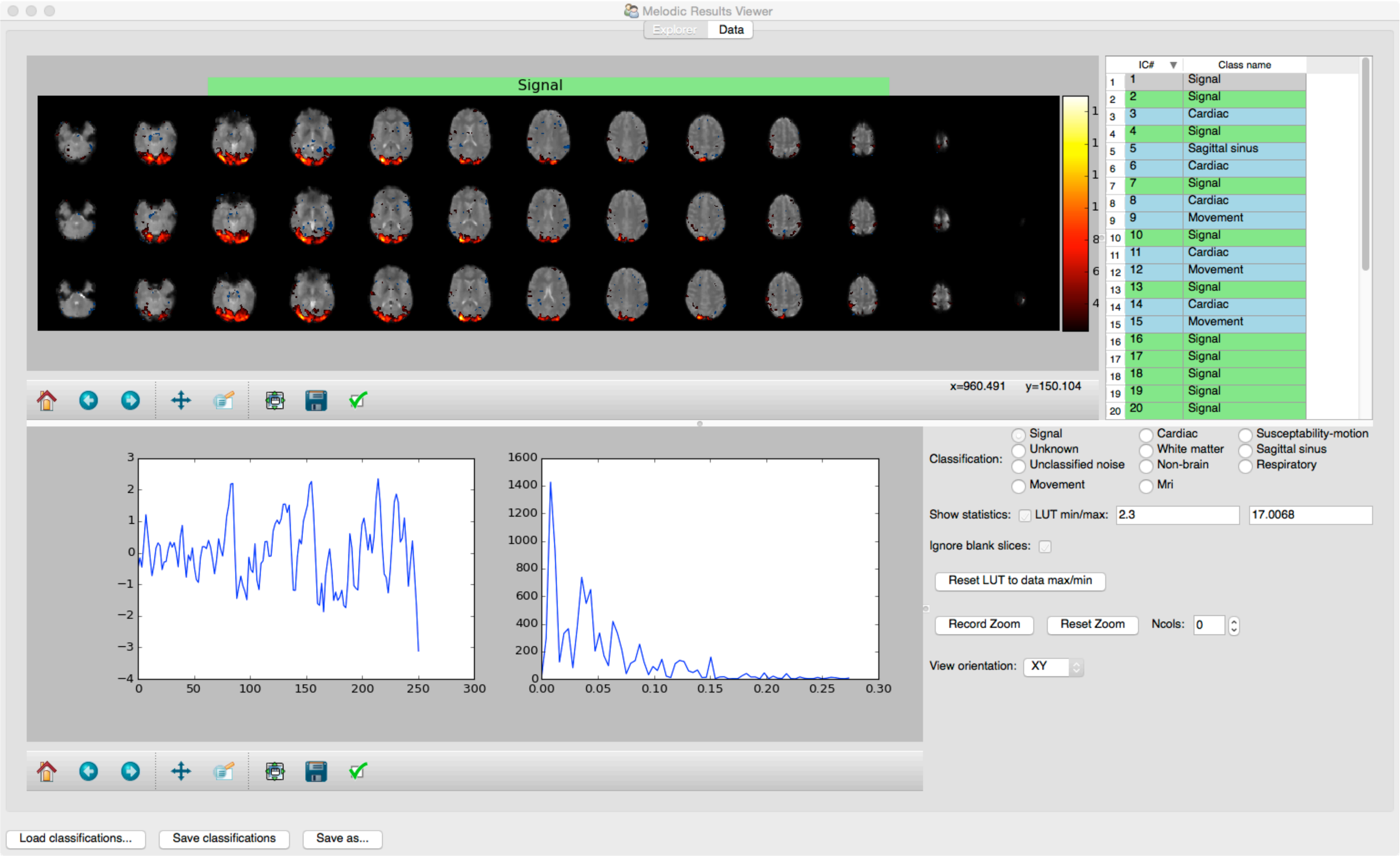

Fig. S1b. Signal ICA component (Medial Visual)

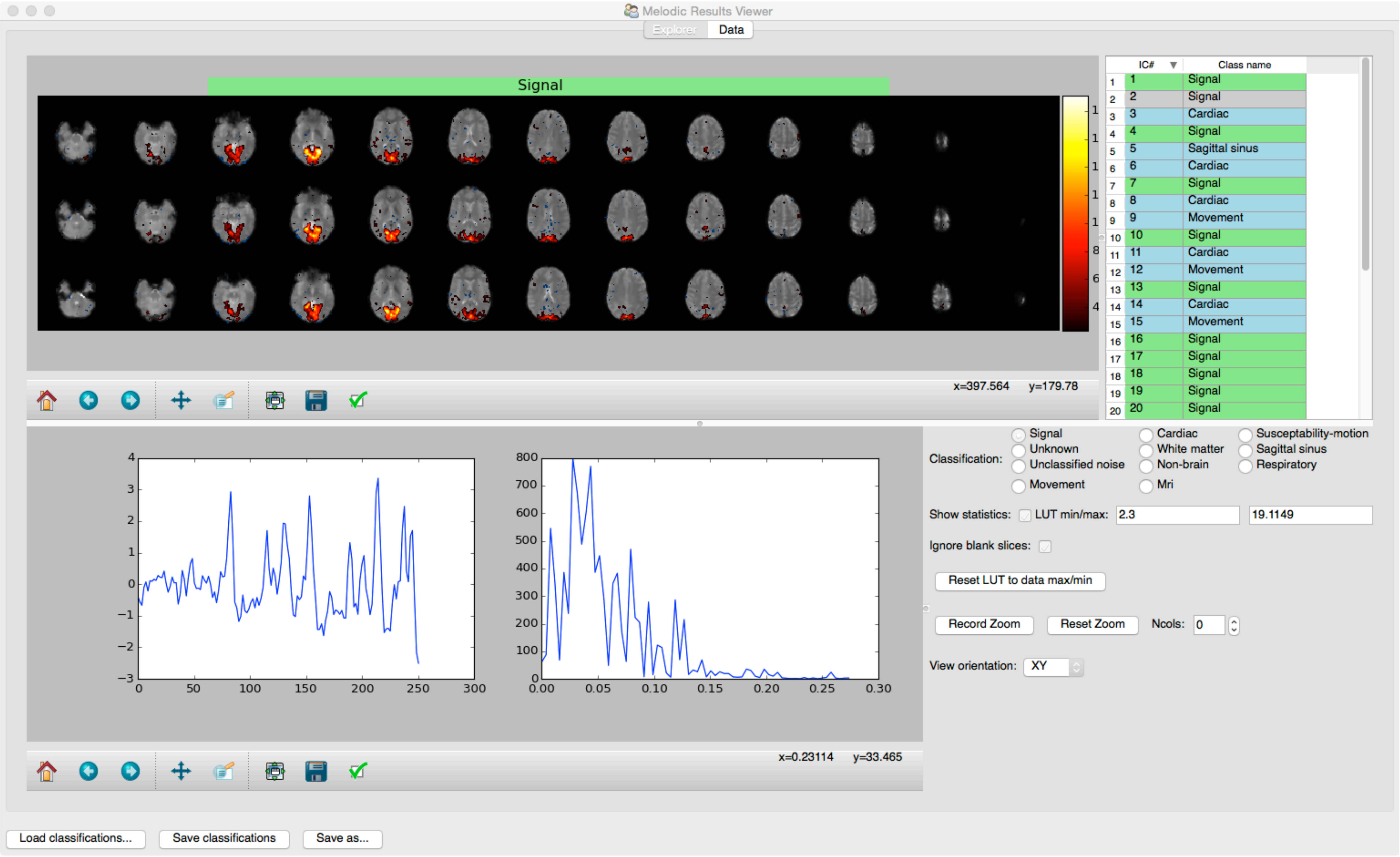

Fig. S1c. Signal ICA component (Default Mode Network)

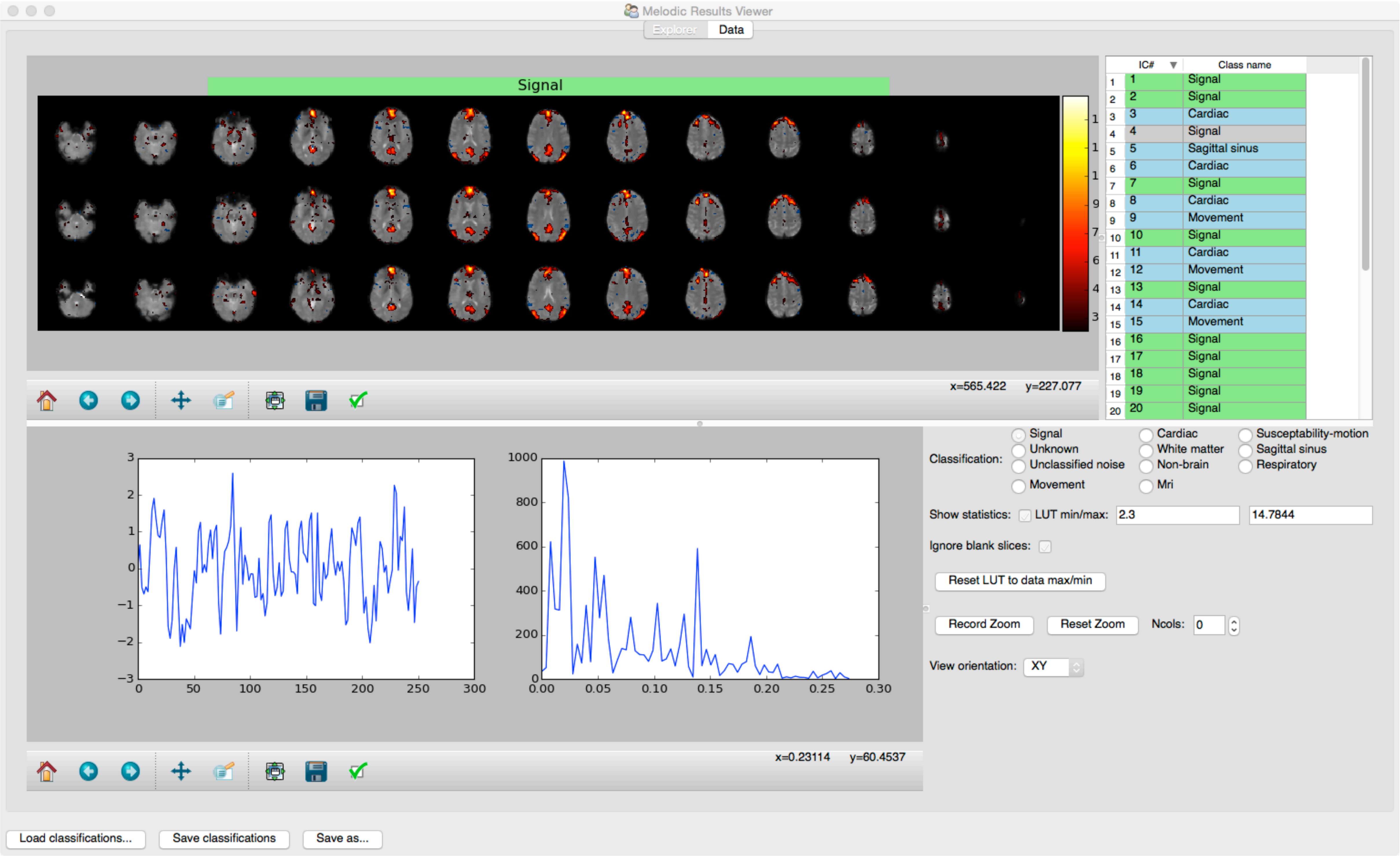

Fig. S1d. Artefactual ICA component (Cardiac)

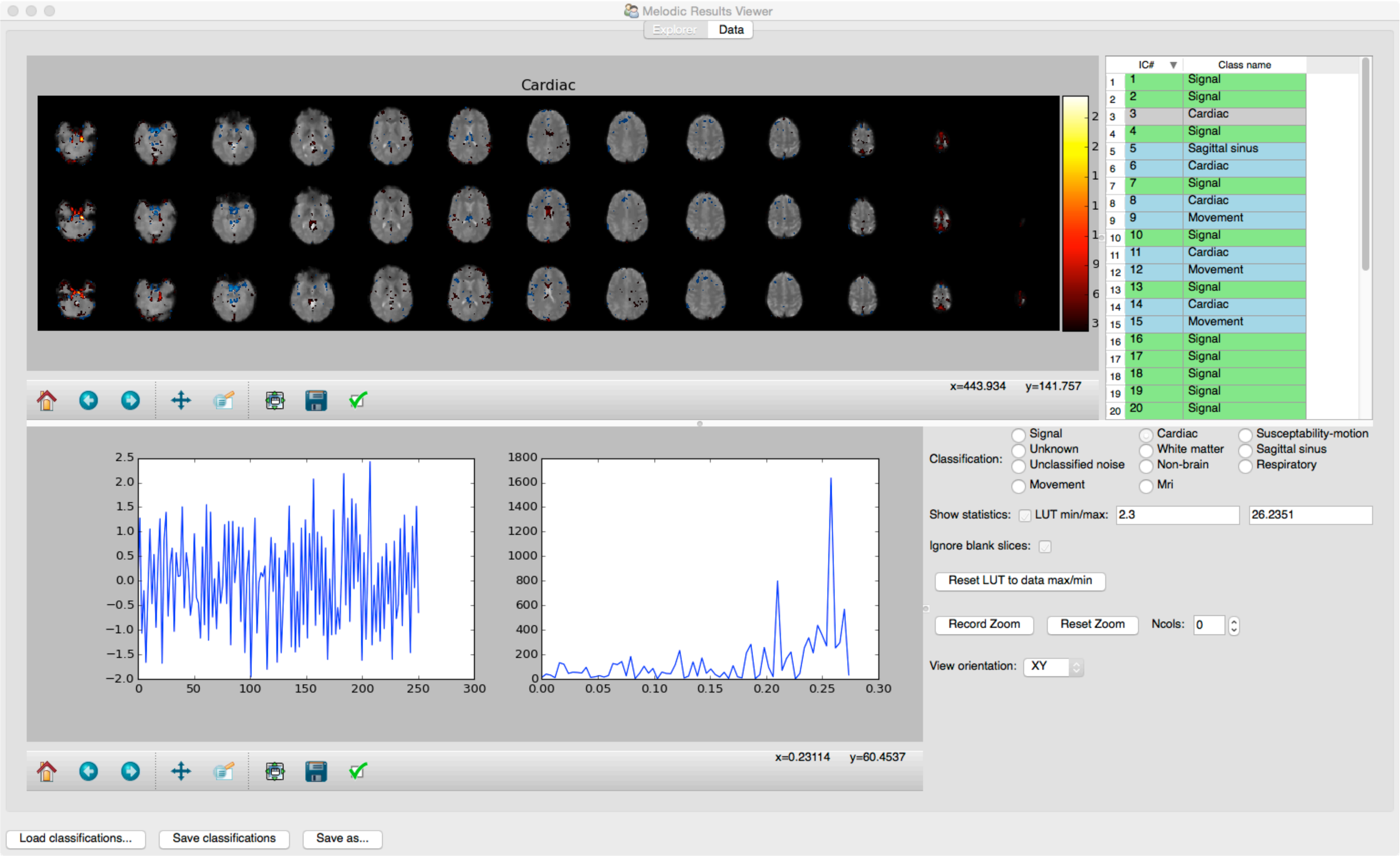

Fig. S1e. Artefactual ICA component (Veins)

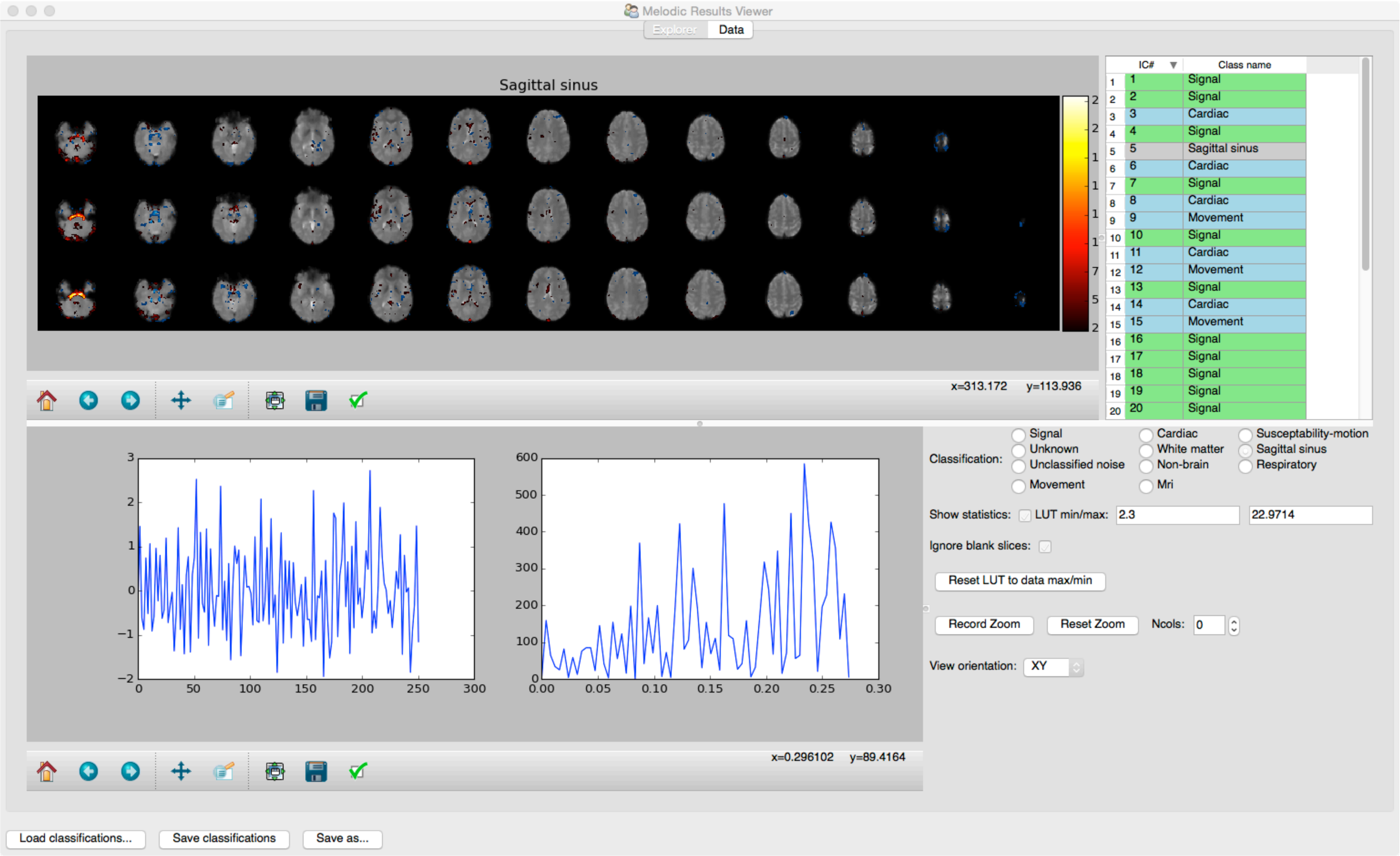

Fig. S1f. Artefactual ICA component (Movement)

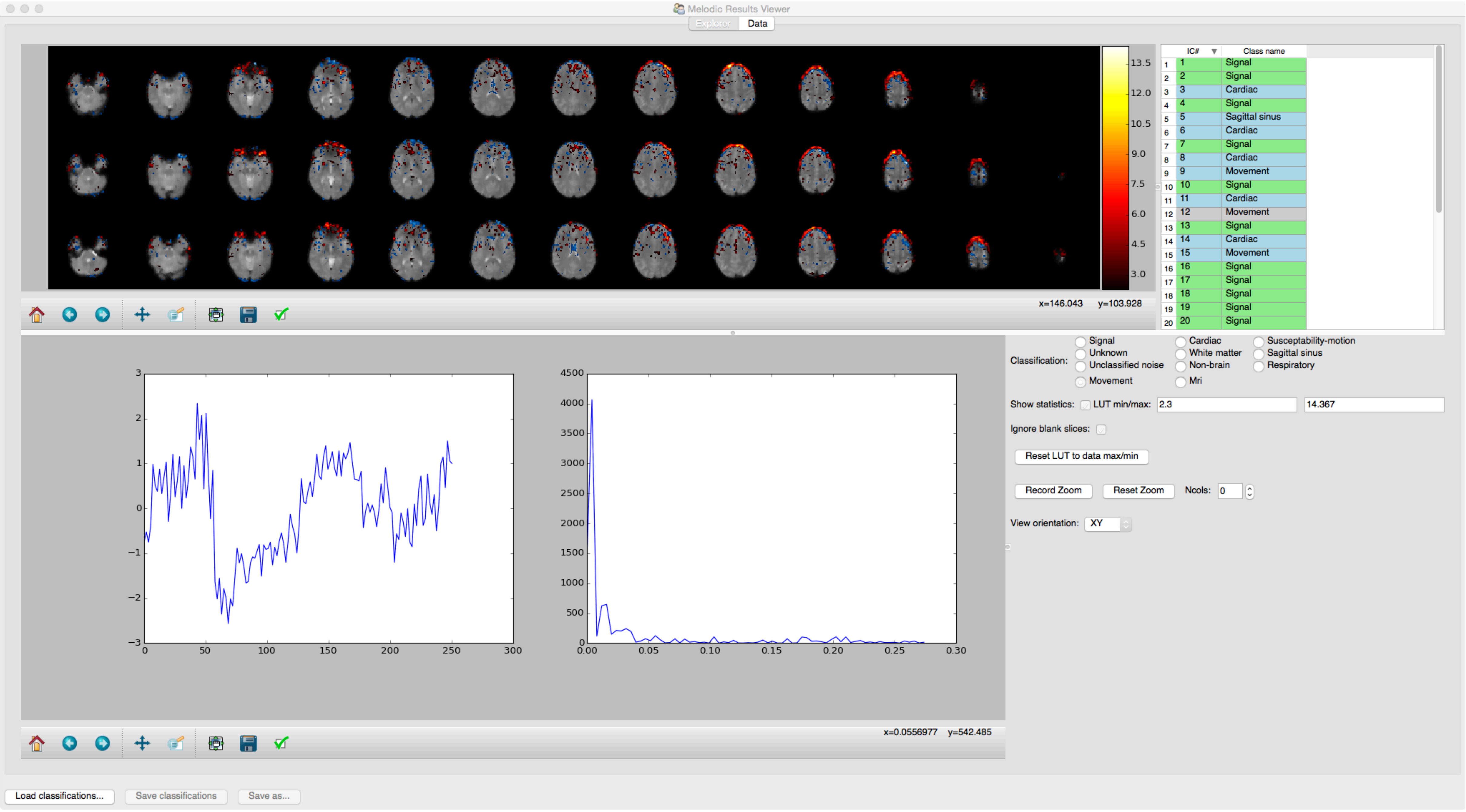

Supplement: S1 Fig — Examples of “good” (panels A, B and C) and “artefactual” (panels D, E and F) components for the first participant. For good components, spectral power lies primarily between 0 and 0.05 Hz and the signal above threshold follows cortical gyrification. For movement-related artefacts, the signal above threshold is essentially at the edges of the brain and the frequencies of the power spectra are disparately distributed. In components due to cardiac pulsation and arterial contribution, the signal above threshold in the spatial maps is essentially located in the ventricles, or following the main arteries (posterior cerebral artery, middle cerebral branches). For components relating to large veins, the signal above threshold in the spatial maps is essentially following the sagittal sinus. Additionally, one may find artefactual components due to MRI acquisition/reconstruction, limited to the white matter or due to susceptibility-motion. For a detailed description of ICA-based artifact removal, see [45, 46]. (PDF) [file pone.0142014.s001.pdf]
